# Influence of Dialysis Membranes on Clinical Outcomes: From History to Innovation

**DOI:** 10.3390/membranes12020152

**Published:** 2022-01-26

**Authors:** Yee-An Chen, Shuo-Ming Ou, Chih-Ching Lin

**Affiliations:** 1 Department of Medicine, Taipei Veterans General Hospital, Taipei 11217, Taiwan; andy560605@gmail.com; 2 Department of Medicine, Division of Nephrology, Taipei Veterans General Hospital, Taipei 11217, Taiwan; 3 School of Medicine, National Yang Ming Chiao Tung University, Taipei 11221, Taiwan; 4 Institute of Clinical Medicine, National Yang Ming Chiao Tung University, Taipei 11221, Taiwan

**Keywords:** cellulose membranes, dialysis membranes, graphene oxide membranes, mixed-matrix membranes, synthetic polymer membranes

## Abstract

Dialysis membranes were traditionally classified according to their material compositions (i.e., as cellulosic or synthetic) and on the basis of the new concept of the sieving coefficient (determined by the molecular weight retention onset and molecular weight cut-off). The advantages of synthetic polymer membranes over cellulose membranes are also described on the basis of their physical, chemical, and structural properties. Innovations of dialysis membrane in recent years include the development of medium cutoff membranes; graphene oxide membranes; mixed-matrix membranes; bioartificial kidneys; and membranes modified with vitamin E, lipoic acid, and neutrophil elastase inhibitors. The current state of research on these membranes, their effects on clinical outcomes, the advantages and disadvantages of their use, and their potential for clinical use are outlined and described.

## 1. Introduction and the History of Dialysis Membranes

**Figure 1:**
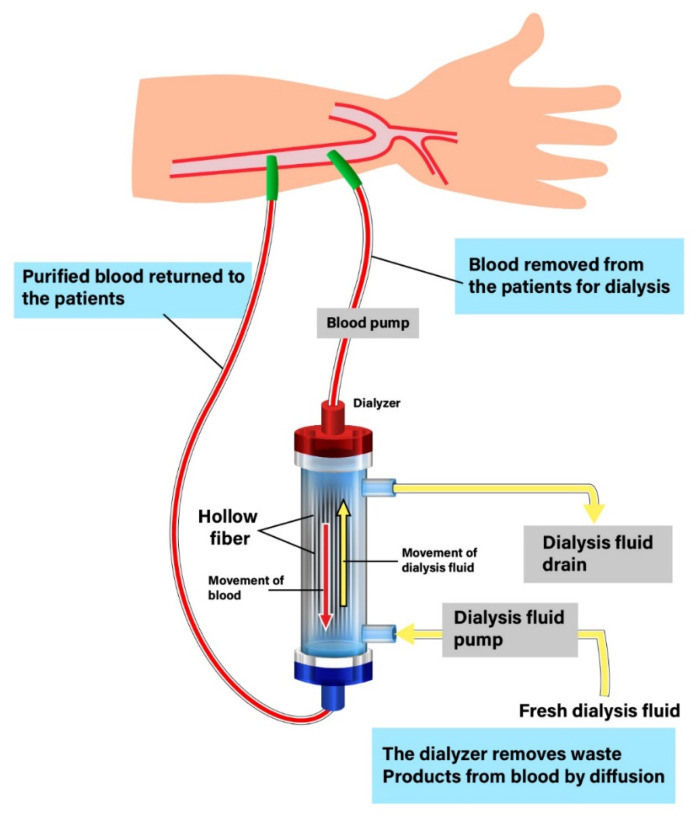
The procedure of hemodialysis. A patient is connected to a dialysis machine and their blood is pumped out via vascular access and filtered using a dialyzer (an artificial kidney containing up to 15,000 hollow fiber membranes). The blood is then pumped back into the patient’s body.

Hemodialysis is an extracorporeal blood-cleansing technique used to remove uremic toxins that accumulate in patients with end-stage renal disease (ESRD). Hemodialysis also removes waters from the body and balances electrolytes, such as potassium, sodium, phosphate, and calcium. Hemodialysis consists of the following steps: A patient is connected to a dialysis machine and their blood is pumped out via vascular access and filtered using a dialyzer (an artificial kidney containing up to 15,000 hollow fiber membranes). The blood is then pumped back into the patient’s body. This therapy can achieve effective removal of small-water soluble toxins (molecular weight, MW < 500 Da) and a small amount of the middle molecules (MW 500–32,000 Da) from the blood of ESRD patients. Solutes and water are removed through semipermeable membranes through different separation mechanisms, such as diffusion and ultrafiltration, which add extra pressure from patient blood and let water and solutes move to the dialysate side. Dialysate is used on the other side of the membrane during dialysis (Figure 1) [1].

A rotating drum kidney was the first membrane configuration used to treat large numbers of patients on hemodialysis (HD) [2]. This device had a 30 m long cellophane tube with an inner diameter of 35 mm that was wrapped in a spiral manner around a cylinder that rotated in a stationary dialysate bath. It lacked a blood pump and could generate only low transmembrane pressures, which affected the ultrafiltration rate. The coil dialyzer was invented to solve these problems [3]. The cellophane tubing comprising the functional unit of this dialyzer, surrounded by a fiberglass screen, was collected in a single coil in a large cylindrical drum to which a recirculating volume of dialysate was delivered. High compartmental blood pressures could be achieved with this device due to the narrowness of the blood channels, but high compartmental resistance was also generated.

In the 1960s, the Kiil dialyzer [4], which employed a parallel blood–dialysate flow configuration, was developed. It consisted of a series of cellophane sheet membranes supported by plastic boards. Diffusive mass transfer efficiency was improved with this device due to the narrowness of the blood channels with material innovation such as the use of a new thin-walled cellulosic membrane. However, all these devices require high compartmental blood volumes and are affected by the inefficient mass transfer characteristics of dialysis membranes, such as the use of concurrent flow and cellophane tubes with wall-thickening and pore size reduction [5,6]. After the 1960s, dialysis membrane innovation shifted to a focus on improving the surface area–to-volume ratio in the blood compartment and reducing boundary layer effects with acceptable end-to-end pressure drops.

## 2. Classification of Dialysis Membranes

Dialysis membranes have traditionally been categorized on the basis of their material composition as cellulosic and synthetic [7]. As synthetic membranes have dominated the market in recent years, with few hospitals using cellulosic membranes, a new membrane classification based on the ultrafiltration coefficient (Kuf) has been proposed. This coefficient, usually expressed in milliliters/hour/millimeters of mercury, is a measure of a membrane’s permeability to water. It is calculated by dividing the ultrafiltration flow rate by the transmembrane pressure. The Kuf value of 12 mL/h/mmHg differentiates low- and high-permeability dialyzers, according to the U.S. Food and Drug Administration [8]. As this classification does not take into consideration small solutes and large molecule clearance, a study of the effect of the dialysis dose and membrane flux in maintenance HD was conducted to improve it [9]. In the new classification, high-flux dialyzers are defined on the basis of two criteria (Kuf > 14 mL/h/mmHg and first-use beta-2 microglobulin (β2m) clearance > 20 mL/min), and low-flux dialyzers are defined on the basis of the single criterion of first-use β2m clearance <10 mL/min. With recognition of the importance of albumin to human health, researchers further modified the classification to consider water permeability, β2m removal, and albumin parameters (Table 1) [10]. High-flux dialysis membranes are defined by a water permeability of 20–40 mL/h/mmHg/m^2^, a β2m sieving coefficient (SC, defined as the ratio of a solute in the ultrafiltrate in comparison to its concentration in the plasma which returns to the patient) of 0.7–0.8, and albumin loss <0.5 g (during 4 h HD); protein-leaking membranes are defined by a water permeability of >40 m/h/mmHg/m^2^, β2m SC of 0.9–1.0, and 2–6 g albumin loss.

The SC describes a membrane’s ability to transport a solute, as a ratio of the solute filtrate and respective solute plasma concentrations. It ranges from 0 (no transport) to 1 (unrestricted transport). Molecular weight retention onset (MWRO) and molecular weight cut-off (MWCO) values were then defined to describe the membrane SC curve. The MWRO is the molecular weight of a given solute at a membrane SC of 0.9, and the MWCO is the molecular weight at an SC of 0.1 (i.e., 10% permeability). The MWRO index provides insight about pore size distribution and the MWCO correlates primarily with the mean pore size. The steepness of the SC versus molecular weight profile is determined largely by the proximity of the values of these two parameters [5].

## 3. Cellulose-Based Membranes

The first cellulose-based dialysis membrane was Cuprophan^®^from Wuppertal, Germany, which was made from cotton. This type of membrane was effective in small solute removal (MW < 500 Da) [11], but had poor outcomes for HD patients comparing with synthetic polymer membranes in additional research into improving the their biocompatibility [12]. Cellulose membranes were enhanced through the chemical masking of the hydroxyl groups such as by their acylation with acetate groups. These modified membranes are composed of cellulose acetate, cellulose diacetate (CDA), and cellulose triacetate (CTA), which were named according to the amount of acetate groups in the each cellulose units. The structural adjustments decreased the majority of free hydroxyl groups on the membrane surface that could bind to complement receptor C3b, the major factor that triggers the complement activation and the cause of adverse events [12]. The CTA dialysis membrane is the most biocompatible in cellulosic membranes nowadays [13].

Another type of synthetically modified cellulose-based dialysis membranes contains aromatic benzyl groups that are fixed on the cellulosic chains and ether bonds in order to make hydrophobic domains [14]. One more type of improvement of cellulosynthetic membrane was Hemophan^®^, in which functional tertiary amines are added during the membrane preparation process. The hydroxyl groups on the membrane surface are adjusted with a large diethylaminoethyl group, which sterically eliminate the reaction between the membrane and blood cells to make a greater biocompatibility (Table 2 and Table 3) [15].

## 4. Synthetic Polymer Membranes

Synthetic polymer membranes are made of polymers such as polysulfone (PSU), polyethersulfone (PES), polymethyl methacrylate, polyester polymer alloy, polyacrylonitrile, polycarbonate, polyamide (PAM), and polyethylene-co-vinyl alcohol. The physicochemical advantages of these membranes over cellulosic membranes include larger pore sizes, better hydraulic permeability, and greater filtration capacities. They also have greater solute removal capacities [24]. Unlike cellulose membranes, which have symmetrical structures and the equally pore size in all layers, synthetic polymer membranes have asymmetrical structures. The outer part of the support layer is formed of a porous skin, which serves as the solute separation barrier. On the other hand, the inner part of this layer is marked by a high density, which decreases from the inside to the outside. The support layer provides mechanical stability and has a microscopically visible sponge-like or finger-type structure [25]. A prospective, randomized, single-center study included 159 patients with ARF requiring HD and revealed no survival difference between meltspun cellulose diacetate, high-flux polysulfone, or low-flux polysulfone [26]. Another RCT trail also included 72 patients treated in intensive care units but did not show significant differences between the low-flux and high-flux groups in terms of survival rate, recovery of renal function, and duration of hemodialysis treatment (Table 2 and Table 3) [27].

Synthetic and cellulose membranes also had different fiber arrangements (Table 4). Cellulose membranes have a wave-like structure in nature, whereas synthetic fibers are crimped to produce a ripple pattern. This difference let synthetic membranes have better blood and dialysate distributions; it also prevents contact or excess packing among fibers and allows for better matching of blood and dialysate flows across all sections of the fiber bundle [28]. However, synthetic polymer membranes also have some disadvantages, including extreme hydrophobicity associated with membrane fouling due to the adhesion of plasma proteins to the membrane surface. This hydrophobicity could cause platelet adhesion, aggregation, and coagulation. In order to improve hydrophobic character, researchers have investigated the use of different synthetic compositions [25]. A Cochrane review of 32 studies found no evidence of a benefit of synthetic versus cellulose/modified cellulose membrane use in the treatment of patients with end-stage renal disease (ESRD) in terms of reduced mortality or dialysis-related adverse symptoms, but showed that the use of synthetic polymer membranes led to a decrease in serum albumin loss and greater reduction of the β2m concentration [29].

PES is the most commonly used materials in dialysis membrane production. It is described as having high oxidative, chemical, and thermal resistance and has appropriate mechanical strength. In addition, it is not changed by sterilization [25]. PES is also used for dialysis membranes due to its high permeability for low-molecular-weight proteins. The main problem with this material is its hydrophobic nature; hydrophilic polymers, most commonly polyvinylpyrrolidone (PVP), are added to it to minimize this problem [28]. PSU-based membranes have some important characteristics, including their high degree of biocompatibility, high permeability for low-molecular-weight proteins, and high retention of endotoxins. A limitation of these membranes is protein accumulation on the membrane surface, which results in reduced flow and changes in membrane selectivity [30], potentially causing immune system activation [31].

## 5. Morphological Difference in Cellulose-Based membranes and Synthetic Polymer Membranes

Most cellulosic membranes are homogeneous and dense, and the entire thickness contributes to the transport resistance for solutes and water. Most synthetic polymeric membranes (except for PMMA, EVAL, and AN-69^®^) are asymmetrical. Physical thickness of synthetic polymeric membranes is thicker (approximately 35 μm) than that of cellulosic membranes (approximately 15 μm) [32]. Mean pore size and pore size distribution substantially affects Kuf and sieving properties of a membrane for different solutes (Table 3). Unlike PSU, the limitation of natural cellulose pore size distribution makes it incompatible for large molecules removal. On the other hand, we could adjust synthetic polymer membranes by changing preparation temperature and adding substances [33].

## 6. Innovation of Membranes

### 6.1. Medium Cutoff Membranes

The main improvement achieved with the shift from high cutoff (HCO, characterized by a substantial increase in water permeability relative to both the high flux and a virgin β2m SC of 1.0) to medium cutoff (MCO) membrane technology was the narrowing of the pore size distribution range. The mean pore radius for MCO membranes is 5 nm (standard deviation, 0.1 nm), which enables more selective removal of solutes with reduced albumin leakage. For comparison, this radius for HCO membranes is 10 nm (standard deviation, 2.0 nm) [34]. A prospective randomized controlled trial (RCT) conducted with 40 patients showed that MCO membranes removed more medium-sized molecules, such as β2m (MW: 11,000 Da), than did high-flux membranes during 3 months of HD [35]. In contrast, MCO-HD did not reduce the serum levels of medium-sized molecules in a prospective cohort of 57 patients followed for 1 year [36]. Thus, further investigation of this capacity of MCO membranes, with long-term follow-up periods, is needed.

An RCT conducted with 48 patients in which MCO-HD was compared with high-flux HD (for 4 weeks with an 8-week extension phase) showed that tumor necrosis factor-α and interleukin-6 mRNA levels decreased, with no difference in cytokine levels (IL-6 MW: 23,718 Da and tumor necrosis factor-α MW: 17,300 Da) under the use of MCO membrane [37]. mRNA levels of such inflammatory factors are intracellular markers and are thus not eliminated during dialysis; the interpretation of this difference requires further study. However, factors including binding to other proteins such as soluble receptors, the formation of multimers, and distribution and re-shift from tissue plasma influence IL serum concentrations and make their measurement and interpretation difficult. MCO-HD may also have an anti-inflammatory effect, but further research is needed to prove this.

A multicenter prospective observational cohort study conducted with 992 patients showed that MCO-HD improved patient-reported quality of life in comparison with patient previous HD experience [38]. However, this study was open-label and may change subjective parameters such as feeling of discomfort during HD and their severity. Albumin (MW: 66,500 Da) loss during long-term MCO-HD is a well-studied issue. In a single-arm study in which MCO membranes were used for 6 months with 87 patients previously on high-flux HD regimens [39] and an RCT conducted with 65 patients on MCO-HD for 6 months [40], serum albumin levels remained stable and thus were not reduced by MCO-HD treatment. A RCT also enrolled 80 patients undergoing thrice-weekly hemodialysis were randomly assigned to receive either expanded hemodialysis (HDx) with medium cutoff (MCO) membranes or online hemodiafiltration, but echocardiographic parameters, cardiovascular mortalities, and all-cause mortalities were the same in both groups. This study showed that MCO membrane was not inferior to online HDF in terms of cardiovascular parameters [41].

### 6.2. Graphene Oxide Membranes

Graphene oxide membranes (GOMs) have some features that make them good membrane candidates, including their high sorption capacity, functional access through covalent and noncovalent interactions, layered structures, amendable interlayer spacing, and expandable dimensions [42]. They have been investigated in in vitro studies. They were found to significantly improve hemocompatibility with little hemolysis, prolonged coagulation times, and low SC5b-9 marker levels [43]. Kidambi et al. [44] fabricated large-area, nanoporous, atomically thin GOMs with size-selective pores (≤1 nm), rapid diffusion, and membrane size selectivity, observing one to two orders of magnitude of improvement in the permeability of small molecules in the molecular weight cutoff range of 0–1000 Da and greater selectivity for the separation of KCl with respect to Allura red dye (≈1 nm, MW: 496 Da) and vitamin B12 (≈1–1.5 nm, MW: 1355 Da) relative to commercial HD membranes. GOMs with greater selective ability may be useful in the clinical setting.

### 6.3. Mixed Matrix Membranes

**Figure 2:**
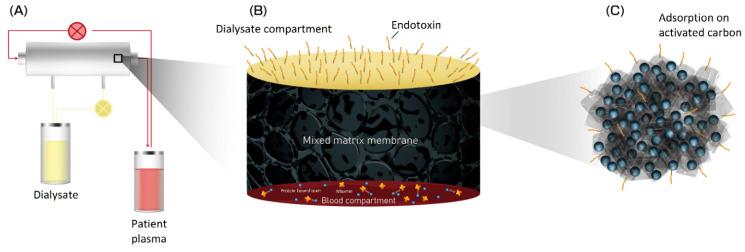
Mixed-matrix membrane (MMM) is made of an inner layer of polyethersulfone (PES)/polyvinylpyrrolidone (PVP) blend and an outer layer of activated carbon (AC) microparticles. It is characterized by the removal of protein-bound toxins from the blood as well as the removal of endotoxins from the dialysate. The endotoxins are adsorbed by activated carbon particles. (**A**) A illustration of gross equipment of mixed-matrix membrane. (**B**) The detail structure of mixed-matrix membrane and we can find endotoxin binded on mixed-matrix membrane (**C**) In molecular level, activated carbon in mixed-matrix membrane grabbed endotoxins and protein-bound toxins.

Mixed-matrix membranes (MMMs) (Figure 2) consist of an inner layer composed of a PES/PVP blend and an outer layer with activated carbon (AC) microparticles. The inner layer confers membrane transport selectivity and prevents contact between the patient’s blood and the adsorbent particles. The outer layer increases the toxin concentration gradient between the blood and dialysate solution, which results in greater removal of uremic solutes by the adsorption toxins [42]. MMMs thus to have good hemocompatibility [45]. They also show more than 100% better ability than commercial dialysis membranes to remove protein-bound uremic toxins, such as indoxyl sulfate (IS, MW: 213 Da) and p-cresyl sulfate (MW: 188 Da) [46]. MMMs remove approximately 10 times more endotoxins (MW: >100,000 Da) from the dialysis fluid than do commercial membranes [47]. Their performance and effects in humans need to be studied, but they have the potential to improve the prognosis of patients on HD due to their ability to remove protein-bound uremic toxins.

### 6.4. Bioartificial Kidneys

**Figure 3:**
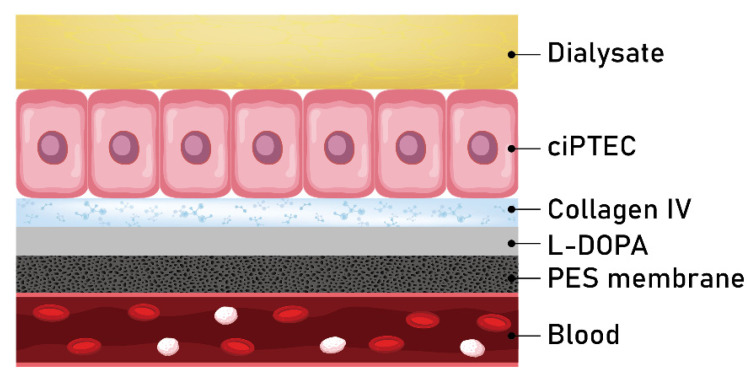
Bioartificial kidneys (BAKs) are membranes that mimic native kidney processes by engaging a monolayer of conditionally immortalized proximal tubule epithelial cells (ciPTECs) cultured on polymeric membranes and collagen IV. Polyethersulfone (PES) membrane layers help ciPTECs avoid direct contact with blood and improve the membrane’s hemocompatibility.

Bioartificial kidneys (BAKs) (Figure 3) are membranes that mimic native kidney processes by engaging a monolayer of conditionally immortalized proximal tubule epithelial cells (ciPTECs) cultured on polymeric membranes and collagen IV [42]. Two in-vitro studies demonstrated that ciPTECs are living cells that perform their functions well: they secrete proinflammatory cytokines and organic cation transporter 2, transport ions well, and are inhibited by H2-receptor antagonists [48,49]. They have been shown to achieve the secretory clearance of human serum albumin-bound uremic toxins that cannot be removed by commercial membranes, including IS (MW: 213 Da) and kynurenic acid (MW: 189 Da), as well as of albumin (MW: 66,500 Da) [49]. In a phase II multicenter open-label RCT conducted with 58 patients with mean sepsis-related organ failure assessment (SOFA) score around 12 and over half of the patients being on ventilator support, the mortality rate at 28 days was lower among patients treated with BAKs and CRRT (33%) than among those treated with CCRT alone (61%); the BAKs also significantly improved survival at 180 days [50]. More studies of the performance and effects of BAKs in humans are needed to confirm that these membranes are suitable for long-term wearable HD applications.

### 6.5. Vitamin E-Modified Membranes

Vitamin E is an important lipophilic antioxidant in human beings. Vitamin E-modified membranes have been designed to decrease oxidative stress in patients on HD. An observational crossover RCT showed that these membranes reduced oxidizing agent processes, such as indoleamine 2,3-dioxygenase-1 activity and nitric oxide formation, in 18 patients on HD [51]. In a multicenter RCT conducted with two parallel groups of 94 patients on HD, theerythropoiesis-stimulating agent resistance index was decreased in the vitamin E–modified membrane group compared with the low-flux synthetic dialyzer group [52]. However, no significant change in the superoxide dismutase or C-reactive protein level or erythropoietin resistance index was observed in another RCT conducted with 80 patients with GSTM1-null genotypes on HD with vitamin E-modified membrane [53]. A systematic review and meta-analysis of 60 studies confirmed that vitamin E-modified membranes significantly decreased the concentrations of IL-6 (MW: 23,718 Da), thiobarbituric acid-reactive substances (MW: 144 Da), and plasma and red blood cell malonylaldehyde (MW: 72 Da), but not those of other oxidizing agents, such as NOx (MW: 30-42 Da) in plasma [54]. A RCT trial also found lower 8-hydroxy 2’-deoxyguanosine level (a surrogate marker of oxidative stress) in leukocyte DNA as compared with the cellulosic group [55].Vitamin E-coated dialyzers were not inferior to heparin-coated dialyzers in no circuit-clotting event (defined as no circuit-blood clot during dialysis leading to premature end of any of the four dialysis sessions) in a multicenter prospective randomized crossover study conducted with 32 adults on long-term HD [56]. In addition, vitamin E-modified membranes have no impact on anemia parameters, lipid profiles, dialysis adequacy, blood pressure, or albumin (MW: 66,500 Da) and uric acid (MW: 168 Da) levels [54]. Studies conducted to date have yielded conflicting results, and additional research on these membranes is needed.

### 6.6. Lipoic Acid-Modified Membranes

Fat-soluble antioxidant lipoic acid has been used as an oral antioxidant supplement to reduce oxidative stress-associated complications in patients on HD [24]. Mahlicli et al. [57] created a bioactive membrane model using lipoic acid and confirmed that it reduced oxidative stress in vitro. In a recent study, PS membranes enriched with α-lipoic acid and α-tocopherol tended to reduce oxidative stress in vivo [58]. These nonhemolytic and hemocompatible membranes may be an antioxidative membrane option that improves the outcomes of patients on HD [58].

### 6.7. Neutrophil Elastase Inhibitor-Modified Membranes

Neutrophil elastase (NE) is a proteinase secreted by neutrophils and macrophages during inflammation that destroys bacteria and host tissue. The reduction of NE activity may reduce inflammation. The ability to ameliorate the negative proteolytic effects of NE in patients with various conditions has been demonstrated, with attenuation of the perioperative inflammatory response and improvement of clinical outcomes in pediatric patients undergoing heart surgery with cardiopulmonary bypass and the improvement of lung function in patients with bronchiectasis [24,59,60]. Grano et al. [61] proposed the immobilization of an NE inhibitor on an HD membrane, and in vitro testing revealed that such membranes effectively reduced the proteolytic activity of NE. However, no in vivo study of NE inhibitor-coated membranes has been performed to date.

## 7. Conclusions

Tracing from history, cellulose-based membranes and synthetic polymer membranes had progressive improvement in recent years. Although synthetic membranes are the most commercialized membrane type, they do not reduce mortality or dialysis-related adverse effects in patients with ESRD. Current innovations in membrane development are focused on the improvement of hemocompatibility and protein-bound uremic toxin removal, as well as the reduction of oxidative stress and albumin loss, with the ultimate aim of reducing mortality among patients on HD (Table 5). Additional clinical studies, however, are needed to further explore the performance and effects of these new membranes.

## Figures and Tables

**Table 1:** The classification and characteristics of dialysis membranes.

	MWRO(Da)	MWCO(Da)	Water Permeability (mL/h/mmHg/m^2^)	Sieving Coefficient		Pore Radius (nm)
				β2m	Albumin	
**Low-flux**	2000–3000	15,000	10–20	-	<0.010	2.0–3.0
**High-flux**	4000–10,000	15,000–16,000	200–400	0.7–0.8	<0.010	3.5–5.5
**Medium cut-off**	10,000–13,000	60,000–100,000	600–850	1	0.008	5.0
**High cut-off**	15,000–20,000	200,000–300,000	1100	1	0.200	8.0–12.0

The membrane classification is based on the ultrafiltration coefficient (Kuf). The cut off value is defined by MWRO and MWCO. Abbreviations: MWRO, molecular weight retention onset; MWCO, molecular weight cut-off; β2m, beta-2 microglobulin.

**Table 2:** Comparison of cellulose-based membranes and synthetic polymer membranes.

Type of Membrane	Designation	Advantages	Disadvantage
Unmodified cellulose	Cuprophan^®^	Better small solute removal and higher HD treatment adequacy compared to modified cellulose and PSU membranes	Higher complement and PMN cell activation Higher risks of penetration of bacterial products from dialysate into blood Not removing medium-sized molecules from the blood
Modified cellulose	Cellulose acetate (CA)	Lower complement activation	Higher neutrophil apoptosis compared to PSU membrane Higher complement activation in comparison to synthetic membranes
	Hemophan^®^	Lower complement activation	Higher pro-inflammatory cytokine production compared to PAM membranes
	Synthetically modified cellulose (SMC)	Lower complement activation	Lower β2m removal compared to synthetic membranes
Synthetic	Polycarbonate (PC)	Naturally hydrophilic character Lower complement activation compared to unmodified cellulose membranes	Higher production of inflammatory markers compared to PAM membranes Higher complement activation compared to PAN and PSU membranes
	Polysulfone (PSU)	Good removal of β2m Lower mortality rate compared to cellulose membranes	Higher neutrophil activation compared to EVAL membranes Increases pro-inflammatory cytokine production
	Polyamide (PAM)	Good removal of β2m	Higher risks of anaphylactic reaction Persistence of slight complement activation
	Polyethersulfone (PES)	Great removal of middle-MW molecules	Protein adsorption on its surface Persistence of immune system activation
	Polyacrylonitrile (PAN)	Adsorption of pro-inflammatory, low–medium-sized proteins and bacterial products Lower neutrophil activation compared to PMMA membranes	Production of bradykinin High risks of anaphylactic reaction compared to other synthetic membranes Persistence of slight complement activation
	Polymethyl methacrylate (PMMA)	Great removal of middle-MW proteins Lower pro-inflammatory cytokine production compared to PS membranes Positive effect on anemia	Persistence of slight complement activation Causes mild leukopenia
	Polyester polymer alloy (PEPA)	Low albumin permeation Good β2m removal	Persistence of low complement activation
	Ethylene-vinyl alcohol copolymer (EVAL)	Naturally hydrophilic character with low protein adsorption Removes high MW molecules Better oxidative stress reduction compared to CA membranes Lower neutrophil activation compared to PS membranes	Mechanical strength is not sufficient to withstand the pressures experienced during HD procedure

Abrreviations: HD, hemodialysis; CA, cellulose acetate; SMC, synthetically modified cellulose; PC, polycarbonate; PSU, polysulfone; PAM, polyamide; β2m, beta-2 microglobulin; PES, polyethersulfone; PAN, polyacrylonitrile; PMMA, polymethyl methacrylate; PEPA, polyester polymer alloy; EVAL, ethylene-vinyl alcohol copolymer; MW, molecular weight.

**Table 3:** Summary of MWCO and pore size in different types of membranes.

Type of Membrane	Designation	MWCO (kDa)	Pore Size	Ref.
Unmodified cellulose	Cuprophan^®^	10 kDa	1.72 nm	[16]
Modified cellulose	Cellulose acetate (CA)	17.6–18.6 kDa	84 nm	[17]
	Hemophan^®^	2 kDa	22 nm	[18]
Synthetic	Polycarbonate (PC)	20 kDa	10–600nm	[19]
	Polysulfone (PSU)	60,000 kDa	5–11 nm	[20]
	Polyamide (PAM)	1000 kDa	-	[20]
	Polyethersulfone (PES)	1–500 kDa	5.12–6.33 nm	[21]
	Polyacrylonitrile (PAN)	100 kDa	5.4 nm	[21]
	Polymethyl methacrylate (PMMA)	55–130 kDa	3.5–5.5 nm	[22]
	Polyester polymer alloy (PEPA)	55–130 kDa	50–500 nm	[22]
	Ethylene-vinyl alcohol copolymer (EVAL)	500 kDa	0.1–0.2 mm	[23]

*Abrreviations:* CA, cellulose acetate; SMC, synthetically modified cellulose; PC, polycarbonate; PSU, polysulfone; PAM, polyamide; PES, polyethersulfone; PAN, polyacrylonitrile; PMMA, polymethyl methacrylate; PEPA, polyester polymer alloy; EVAL, ethylene-vinyl alcohol copolymer; Ref., references.

**Table 4:** Comparison of SEM picture between cellulose-based and synthetic polymer membranes [5].

Cellulose-Based (Cuprophan)	Synthetic (Polysulfone)
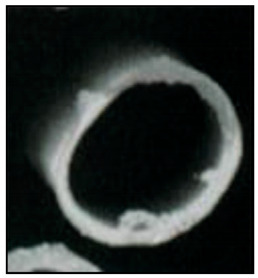	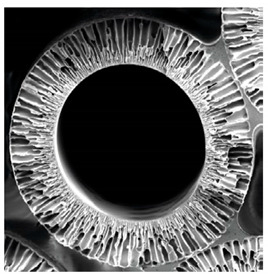
Natural polymer	Synthetic polymer
Homogeneous	Asymmetry
Hydrophilic (hydrogel)	Hydrophobic structure
Low hydraulic permeability	High hydraulic permeability
Low sieving properties	High sieving properties
Prevalent use in hemodialysis	Exclusively used for hemofiltration

Adapted by permission from Springer Nature: Claudio Ronco, William R Clark. Haemodialysis membranes. Nat Rev Nephrol. 2018 Jun;14(6):394-410. License Number: 5235610501855. *Abbreviations*: SEM, scanning electron microscopy.

**Table 5:** Summary of membrane innovation.

Membrane Type	MWCO (kDa)	Advantage	Disadvantage	Ref.
Medium cutoff membranes	60–100	Increases water permeability relative to both the high-flux and a virgin β2m SC of 1.0 May have an anti-inflammatory effect Decreases extra albumin loss compare with high-flux membranes	Cannot reduce the serum levels of medium-sized molecules in long-term follow-up	RCTs: [35,37,39,40,41] Observational study: [36,38]
Graphene oxide membranes	1–3	Improves the permeability of small molecules (MW: 0–1000 Da) with size-selective pores (≤1 nm)	Still in in vitro studies	In vitro study: [43,44]
Mixed-matrix membranes	47	Removes more uremic solutes by absorbing toxins Removes about 10 times more endotoxins than conventional membranes	Still in in vitro studies	In vitro study: [45,46,47]
Bioartificial kidneys	10–30	Achieves the secretory clearance of human serum albumin-bound uremic toxins	Concern with long term use	RCTs: [50] In vitro study: [48,49]
Vitamin E-modified membranes	10–300	Not inferior to heparin-coated dialyzers in anti-coagulation May decrease oxidative stress	Have no impact on anemia parameters, lipid profiles, dialysis adequacy, blood pressure, or albumin	RCTs: [51,52,53,55,56] Meta-analysis: [54]
Lipoic acid-modified membranes	10	Reduces oxidative stress in in vitro study	Still in in vitro studies	In vitro study: [57,58]
Neutrophil elastase inhibitor modified membranes	2	Effectively reduces the proteolytic activity of neutrophil elastase	Lack of in vivo study of NE inhibitor-coated membranes	In vitro study: [61]

Abbreviations: MWCO, molecular weight cut-off; β2m, beta-2 microglobulin; SC, sieving coefficient; MW, molecular weight; NE, neutrophil elastase; RCT, randomized controlled trial; Ref., references.

## Data Availability

Not applicable.

## References

[1] Blowey D.L., Alon U.S. (2005). Dialysis principles for primary health-care providers. Clin. Pediatr. Phila.

[2] Kolff W.J., Berk H.T., Welle N.M., van der Ley A.J., van Dijk E.C., van Noordwijk J. (1997). The artificial kidney: A dialyser with a great area. 1944. J. Am. Soc. Nephrol..

[3] Kolff W.J., Watschinger B., Vertes V. (1956). Results in patients treated with the coil kidney (disposable dialyzing unit). J. Am. Med. Assoc..

[4] Kiil F. (1960). Development of a parallel-flow artificial kidney in plastics. Acta Chir. Scand. Suppl..

[5] Ronco C., Clark W.R. (2018). Haemodialysis membranes. Nat. Rev. Nephrol..

[6] Twardowski Z.J. (2008). History of hemodialyzers' designs. Hemodial. Int..

[7] Zhu L., Liu F., Yu X., Xue L. (2015). Poly(Lactic Acid) Hemodialysis Membranes with Poly(Lactic Acid)-block-Poly(2-Hydroxyethyl Methacrylate) Copolymer As Additive: Preparation, Characterization, and Performance. ACS Appl. Mater. Interfaces.

[8] FDA, US Food and Drug Administration (1998). Guidance for Thecontent of Premarket Notifications for Conventional and High Per-Meability Hemodialyzers.

[9] Locatelli F. (2003). Effect of dialysis dose and membrane flux in maintenance hemodialysis. N. Engl. J. Med..

[10] Ward R.A. (2005). Protein-leaking membranes for hemodialysis: A new class of membranes in search of an application?. J. Am. Soc. Nephrol..

[11] MacLeod A., Daly C., Khan I., Vale L., Campbell M., Wallace S., Cody J., Donaldson C., Grant A. (2001). Comparison of cellulose, modified cellulose and synthetic membranes in the haemodialysis of patients with end-stage renal disease. Cochrane Database Syst. Rev..

[12] Clark W.R., Hamburger R.J., Lysaght M.J. (1999). Effect of membrane composition and structure on solute removal and biocompatibility in hemodialysis. Kidney Int..

[13] Schaefer R.M., Hörl W.H., Kokot K., Heidland A. (1987). Enhanced biocompatibility with a new cellulosic membrane: Cuprophan versus Hemophan. Blood Purif..

[14] Hoenich N.A., Woffindin C., Stamp S., Roberts S.J., Turnbull J. (1997). Synthetically modified cellulose: An alternative to synthetic membranes for use in haemodialysis?. Biomaterials.

[15] Subramanian S., Venkataraman R., Kellum J.A. (2002). Influence of dialysis membranes on outcomes in acute renal failure: A meta-analysis. Kidney Int..

[16] Drioli E., Giorno L., Fontananova E. (2017). Comprehensive Membrane Science and Engineering 2.13—Progress in the Development of Membranes for Kidney-Replacement Therapy.

[17] Janeca A., Rodrigues F.S.C., Gonçalves M.C., Faria M. (2021). Novel Cellulose Acetate-Based Monophasic Hybrid Membranes for Improved Blood Purification Devices: Characterization under Dynamic Conditions. Membranes.

[18] Ismail A.F., Rahman M.A., Othman M.H., Matsuura T. (2018). Membrane Separation Principles and Applications.

[19] Ao X., Stenken J.A. (2003). Water-soluble cyclodextrin polymers for enhanced relative recovery of hydrophobic analytes during microdialysis sampling. Analyst.

[20] Abdelrasoul A., Doan H., Lohi A., Cheng C.H. (2018). The effect of contaminated particle sphericity and size on membrane fouling in cross flow ultrafiltration. Environ. Technol.

[21] Dang H., Narbaitz R., Matsuura T., Khulbe K. (2006). A Comparison of Commercial and Experimental Ultrafiltration Membranes via Surface Property Analysis and Fouling Tests. Water Qual. Res. J. Can..

[22] A Clinical Update on Dialyzer Membranes State-of-the-Art Considerations for Optimal Care in Hemodialysis. https://www.semanticscholar.org/paper/A-Clinical-Update-on-Dialyzer-Membranes-for-Optimal/17673ccd07a90b45f3d68d178be3031ba2bff55a.

[23] Huang L., Ye H., Yu T., Zhang X., Zhang Y., Zhao L., Xin Q., Wang S., Ding X., Li H. (2018). Similarly sized protein separation of charge-selective ethylene-vinyl alcohol copolymer membrane by grafting dimethylaminoethyl methacrylate. J. Appl. Polym. Sci..

[24] Kohlová M., Amorim C.G., Araújo A., Santos-Silva A., Solich P., Montenegro M. (2019). The biocompatibility and bioactivity of hemodialysis membranes: Their impact in end-stage renal disease. J. Artif. Organs.

[25] Olczyk P., Małyszczak A., Kusztal M. (2018). Dialysis membranes: A 2018 update. Polim. Med..

[26] Gastaldello K., Melot C., Kahn R.J., Vanherweghem J.L., Vincent J.L., Tielemans C. (2000). Comparison of cellulose diacetate and polysulfone membranes in the outcome of acute renal failure. A prospective randomized study. Nephrol. Dial. Transplant..

[27] Ponikvar J.B., Rus R.R., Kenda R.B., Bren A.F., Ponikvar R.R. (2001). Low-flux versus high-flux synthetic dialysis membrane in acute renal failure: Prospective randomized study. Artif. Organs.

[28] Ronco C., Levin N., Brendolan A., Nalesso F., Cruz D., Ocampo C., Kuang D., Bonello M., De Cal M., Corradi V. (2006). Flow distribution analysis by helical scanning in polysulfone hemodialyzers: Effects of fiber structure and design on flow patterns and solute clearances. Hemodial. Int..

[29] Macleod A.M., Campbell M., Cody J.D., Daly C., Donaldson C., Grant A., Khan I., Rabindranath K.S., Vale L., Wallace S. (2005). Cellulose, modified cellulose and synthetic membranes in the haemodialysis of patients with end-stage renal disease. Cochrane Database Syst. Rev..

[30] Su B.-H., Shi Y., Fu P., Tao Y., Nie S., Zhao C.-S. (2012). Clinical evaluation of polyethersulfone high-flux hemodialysis membrane compared to other membranes. J. Appl. Polym. Sci..

[31] Wenten I.G., Aryanti P.T.P., Khoiruddin K., Hakim A.N., Himma N.F. (2016). Advances in Polysulfone-Based Membranes for Hemodialysis. J. Membr. Sci. Res..

[32] Sakurai A. (2015). Dialysis Membranes—Physicochemical Structures and Features, Updates in Hemodialysis.

[33] Bowry S.K. (2002). Dialysis membranes today. Int. J. Artif. Organs.

[34] Reis T., Anwar S., Neves F., Ronco C. (2021). Disruptive technologies for hemodialysis: Medium and high cutoff membranes. Is the future now?. J. Bras. Nefrol..

[35] Belmouaz M., Bauwens M., Hauet T., Bossard V., Jamet P., Joly F., Chikhi E., Joffrion S., Gand E., Bridoux F. (2020). Comparison of the removal of uraemic toxins with medium cut-off and high-flux dialysers: A randomized clinical trial. Nephrol. Dial. Transplant..

[36] Cho N.J., Park S., Islam M.I., Song H.Y., Lee E.Y., Gil H.W. (2019). Long-term effect of medium cut-off dialyzer on middle uremic toxins and cell-free hemoglobin. PLoS ONE.

[37] Zickler D., Schindler R., Willy K., Martus P., Pawlak M., Storr M., Hulko M., Boehler T., Glomb M.A., Liehr K. (2017). Medium Cut-Off (MCO) Membranes Reduce Inflammation in Chronic Dialysis Patients-A Randomized Controlled Clinical Trial. PLoS ONE.

[38] Alarcon J.C., Bunch A., Ardila F., Zuñiga E., Vesga J.I., Rivera A., Sánchez R., Sanabria R.M. (2021). Impact of Medium Cut-Off Dialyzers on Patient-Reported Outcomes: COREXH Registry. Blood Purif..

[39] Krishnasamy R., Hawley C.M., Jardine M.J., Roberts M.A., Cho Y., Wong M., Heath A., Nelson C.L., Sen S., Mount P.F. (2020). A tRial Evaluating Mid Cut-Off Value Membrane Clearance of Albumin and Light Chains in HemoDialysis Patients: A Safety Device Study. Blood Purif..

[40] Weiner D.E., Falzon L., Skoufos L., Bernardo A., Beck W., Xiao M., Tran H. (2020). Efficacy and Safety of Expanded Hemodialysis with the Theranova 400 Dialyzer: A Randomized Controlled Trial. Clin. J. Am. Soc. Nephrol..

[41] Lee Y., Jang M.J., Jeon J., Lee J.E., Huh W., Choi B.S., Park C.W., Chin H.J., Kang C.L., Kim D.K. (2021). Cardiovascular Risk Comparison between Expanded Hemodialysis Using Theranova and Online Hemodiafiltration (CARTOON): A Multicenter Randomized Controlled Trial. Sci. Rep..

[42] Basile C., Davenport A., Mitra S., Pal A., Stamatialis D., Chrysochou C., Kirmizis D. (2021). Frontiers in hemodialysis: Innovations and technological advances. Artif. Organs.

[43] Modi A., Verma S.K., Bellare J. (2018). Graphene oxide-doping improves the biocompatibility and separation performance of polyethersulfone hollow fiber membranes for bioartificial kidney application. J. Colloid. Interface Sci..

[44] Kidambi P.R., Jang D., Idrobo J.C., Boutilier M.S.H., Wang L., Kong J., Karnik R. (2017). Nanoporous Atomically Thin Graphene Membranes for Desalting and Dialysis Applications. Adv. Mater..

[45] Geremia I., Pavlenko D., Maksymow K., Rüth M., Lemke H.D., Stamatialis D. (2020). Ex vivo evaluation of the blood compatibility of mixed matrix haemodialysis membranes. Acta Biomater..

[46] Pavlenko D., van Geffen E., van Steenbergen M.J., Glorieux G., Vanholder R., Gerritsen K.G., Stamatialis D. (2016). New low-flux mixed matrix membranes that offer superior removal of protein-bound toxins from human plasma. Sci. Rep..

[47] Geremia I., Bansal R., Stamatialis D. (2019). In vitro assessment of mixed matrix hemodialysis membrane for achieving endotoxin-free dialysate combined with high removal of uremic toxins from human plasma. Acta Biomater..

[48] Jansen J., De Napoli I.E., Fedecostante M., Schophuizen C.M., Chevtchik N.V., Wilmer M.J., van Asbeck A.H., Croes H.J., Pertijs J.C., Wetzels J.F. (2015). Human proximal tubule epithelial cells cultured on hollow fibers: Living membranes that actively transport organic cations. Sci. Rep..

[49] Chevtchik N.V., Mihajlovic M., Fedecostante M., Bolhuis-Versteeg L., Sastre Toraño J., Masereeuw R., Stamatialis D. (2018). A bioartificial kidney device with polarized secretion of immune modulators. J. Tissue Eng. Regen. Med..

[50] Tumlin J., Wali R., Williams W., Murray P., Tolwani A.J., Vinnikova A.K., Szerlip H.M., Ye J., Paganini E.P., Dworkin L. (2008). Efficacy and safety of renal tubule cell therapy for acute renal failure. J. Am. Soc. Nephrol..

[51] Sepe V., Gregorini M., Rampino T., Esposito P., Coppo R., Galli F., Libetta C. (2019). Vitamin e-loaded membrane dialyzers reduce hemodialysis inflammaging. BMC Nephrol..

[52] Locatelli F., Andrulli S., Viganò S.M., Concetti M., Urbini S., Giacchino F., Broccoli R., Aucella F., Cossu M., Conti P. (2017). Evaluation of the Impact of a New Synthetic Vitamin E-Bonded Membrane on the Hypo-Responsiveness to the Erythropoietin Therapy in Hemodialysis Patients: A Multicenter Study. Blood Purif..

[53] Djuric P., Suvakov S., Simic T., Markovic D., Jerotic D., Jankovic A., Bulatovic A., Tosic Dragovic J., Damjanovic T., Marinkovic J. (2020). Vitamin E-Bonded Membranes Do Not Influence Markers of Oxidative Stress in Hemodialysis Patients with Homozygous Glutathione Transferase M1 Gene Deletion. Toxins.

[54] D'Arrigo G., Baggetta R., Tripepi G., Galli F., Bolignano D. (2017). Effects of Vitamin E-Coated versus Conventional Membranes in Chronic Hemodialysis Patients: A Systematic Review and Meta-Analysis. Blood Purif..

[55] Tarng D.C., Huang T.P., Liu T.Y., Chen H.W., Sung Y.J., Wei Y.H. (2000). Effect of vitamin E-bonded membrane on the 8-hydroxy 2′-deoxyguanosine level in leukocyte DNA of hemodialysis patients. Kidney Int..

[56] Islam M.S., Hassan Z.A., Chalmin F., Vido S., Berrada M., Verhelst D., Donnadieu P., Moranne O., Esnault V.L. (2016). Vitamin E-Coated and Heparin-Coated Dialyzer Membranes for Heparin-Free Hemodialysis: A Multicenter, Randomized, Crossover Trial. Am. J. Kidney Dis..

[57] Mahlicli F.Y., Altinkaya S.A. (2014). Immobilization of alpha lipoic acid onto polysulfone membranes to suppress hemodialysis induced oxidative stress. J. Membr. Sci..

[58] Kohlová M., Rocha S., Gomes Amorim C., de Nova Araújo A., Santos-Silva A., Solich P., Branco da Silva Montenegro M.C. (2020). Doping Polysulfone Membrane with Alpha-Tocopherol and Alpha-Lipoic Acid for Suppressing Oxidative Stress Induced by Hemodialysis Treatment. Macromol. Biosci..

[59] Kohira S., Oka N., Inoue N., Itatani K., Hanayama N., Kitamura T., Fujii M., Takeda A., Oshima H., Tojo K. (2013). Effect of the neutrophil elastase inhibitor sivelestat on perioperative inflammatory response after pediatric heart surgery with cardiopulmonary bypass: A prospective randomized study. Artif. Organs.

[60] Stockley R., De Soyza A., Gunawardena K., Perrett J., Forsman-Semb K., Entwistle N., Snell N. (2013). Phase II study of a neutrophil elastase inhibitor (AZD9668) in patients with bronchiectasis. Respir. Med..

[61] Grano V., Tasco G., Casadio R., Diano N., Portaccio M., Rossi S., Bencivenga U., Compiani M., De Maio A., Mita D.G. (2004). Reduction of active elastase concentration by means of immobilized inhibitors: A novel therapeutic approach. Biotechnol. Prog..

